# Towards Transfer Learning Techniques—BERT, DistilBERT, BERTimbau, and DistilBERTimbau for Automatic Text Classification from Different Languages: A Case Study

**DOI:** 10.3390/s22218184

**Published:** 2022-10-26

**Authors:** Rafael Silva Barbon, Ademar Takeo Akabane

**Affiliations:** Postgraduate Program in Urban Infrastructure Systems and Telecommunication Networks Management, Centre for Exact Sciences, Technology and the Environment (CEATEC), Pontifical Catholic University of Campinas (PUC-Campinas), 1516 Professor Dr. Euryclides de Jesus Zerbini, Campinas 13086900, SP, Brazil

**Keywords:** big data, pre-trained model, BERT, DistilBERT, BERTimbau, DistilBERTimbau, transformer-based machine learning

## Abstract

The Internet of Things is a paradigm that interconnects several smart devices through the internet to provide ubiquitous services to users. This paradigm and Web 2.0 platforms generate countless amounts of textual data. Thus, a significant challenge in this context is automatically performing text classification. State-of-the-art outcomes have recently been obtained by employing language models trained from scratch on corpora made up from news online to handle text classification better. A language model that we can highlight is BERT (Bidirectional Encoder Representations from Transformers) and also DistilBERT is a pre-trained smaller general-purpose language representation model. In this context, through a case study, we propose performing the text classification task with two previously mentioned models for two languages (English and Brazilian Portuguese) in different datasets. The results show that DistilBERT’s training time for English and Brazilian Portuguese was about 45% faster than its larger counterpart, it was also 40% smaller, and preserves about 96% of language comprehension skills for balanced datasets.

## 1. Introduction

It is known that the support of computational systems is in several areas of knowledge, be it in the human, exact, and biological areas. Consequently, this contributes to the accelerated increase in the generation, consumption, and transmission of data in the global network. According to the study by the Statista Research Department [1], in 2018, the total amount of data created, captured, and consumed in the world was 33 zettabytes (ZB)—equivalent to 33 trillion gigabytes. Already in 2020 it has grown to 59 ZB and is expected to reach 175 ZB by 2025.

In the Internet of Things (IoT) context, we know that these devices (e.g., virtual assistants) are connected to the Internet and generate large amounts of data. On the other hand, we also have Web 2.0 platforms, e.g., social networks, micro-blogs, and all these types of websites with massive amounts of textual information available online. It is worth mentioning that the data generated by these devices and websites are growing faster and faster. An important point worth mentioning is that the information generated from a large amount of text/data generated by users for many entrepreneurs or public agents is vital for maintaining their business. This way, one can exploit this constant and continuous feedback on a particular subject/product through these data. Due to the ever-increasing volume of online text data, the text classification task is more necessary than ever. In this context, text classification (automatically classifying textual) is an essential task.

Automatic text classification can be described as a task that automatically categorizes group documents into one or more predefined classes according to their topics. Thereby, the primary objective of text classification is to extract information from textual resources. The text classification task is the basic module for many NLP (natural language processing) applications. However, this necessitates the presence of efficient and flexible methods to access, organize, and extract useful information from different data sources. These methods can include text classification [2,3,4], information retrieval [5,6], summarization [7,8], text clustering [9,10], and others, collectively named text mining [2,4,6].

Many works are available in the literature on text classification tasks using various neural network models. Some typical works include convolutional neural network (CNN) models [11,12], attentional models [13,14], adversarial models [3], and recurrent neural network (RNN) models [13], which particularly outperform many statistics-based models. The previously mentioned works represent text based on words, i.e., word vectors pre-trained over a large-scale corpus are usually used as the sequence features. Such vectors are usually trained via the word2vec tool [15] or Glove [16,17] algorithm based on the presumption that similar words tend to appear in similar contexts.

In recent years, to avoid specific structures and significantly decrease the parameters to be learned from scratch, as is done in the models presented above, some researchers have contributed in another direction, highlighting the pre-training models for general language and fine-tuning them to downstream tasks. Another problem with traditional NLP approaches worth mentioning is the issue of multilingualism [18]. The Open AI group (https://openai.com/, accessed on 13 July 2022) proposes the GPT (Generative Pre-trained Transformer) using a left-to-right multi-layer Transformer architecture to learn the general language presentations from a large-scale corpus to deal with the abovementioned problems [19]. Later, Google, inspired by GPT, presented a new language representation called BERT (Bidirectional Encoder Representations from Transformers) [20]. BERT is a state-of-the-art language representation model designed to pre-train deep bidirectional representations from unlabeled text and is fine-tuned using labeled text for different NLP tasks [20]. A smaller, faster, and lighter version of BERT architecture, well-known as DistilBERT, was implemented by the HuggingFace team (https://github.com/huggingface/transformers, accessed on 13 July 2022).

This work aimed to examine an extensive dataset from different contexts, including datasets from different languages, specifically English and Brazilian Portuguese, to analyze the performance of the two models (BERT and DistilBERT). To do this, we first fine-tuned BERT and DistilBERT, then the aggregating layer was utilized as the text embedding, and then we compared the two models with several selected datasets. As a general result, we can highlight that the DistilBERT is nearly 40% smaller and around 45% faster than its larger counterpart. Yet, it preserves around 96% of language comprehension skills for both English and Brazilian Portuguese for balanced datasets.

The main contributions of the paper are as follows:

- We compare BERT and DistilBERT, demonstrating how the Light Transformer model can be very close in effectiveness compared to its larger model for different languages;
- We compared models Transformer (BERT) and Light Transformer (DistilBERT) for both English and Brazilian Portuguese.

The rest of the document is organized as follows: Section 2 presents a short summary of the necessary concepts to understand this work, while Section 3 presents the method and hyperparameter configuration for automatic text classification. The case study of this work is presented in Section 4, and the results are presented in Section 5. Thereafter, in Section 6, we discuss the performance of the two models (BERT and DistilBERT) in the different datasets used. Finally, Section 7 concludes with a discussion and recommendations for future work.

## 2. Theoretical Foundation

This section presents the theoretical foundation for a better understanding of the work. In Section 2.1, the Transformer architecture is described, while in Section 2.2, Bidirectional Encoder Representations from Transformers (BERT) is described. In Section 2.3, the comprehension models are presented, and finally, in Section 2.4, the BERTimbau model is introduced.

### 2.1. Transformer Architecture

It is essential to review two concepts: (i) encoder–decoder [21]; and (ii) attention [22] configurations to understand the Transformer architecture. The first concept refers to the type of training adopted to produce embeddings from input tokens.

The second is a technique to circumvent a common problem in sequential architectures applied to natural language processing problems (e.g., recurrent networks [23]). Sequential networks attempt to map the relationship between a token in the target sequence with the source sequence tokens. However, a token in the target sequence may be closer to one or more tokens in the source sequence rather than the entire source sequence. In this way, the network used to generate the representation of the tokens ends up encoding information that may not be relevant to the problem at hand. This problem occurs mainly when the input sequence is long and rich in information and selecting the essential passages is not possible.

In a few words, the idea of the attention mechanism is to make this selection explicit, consisting of a neural layer created exclusively to understand this context relationship between tokens. In this context, Vaswani et al. [19] proposed the Transformer architecture, an encoder–decoder network based on parallelization of the attention mechanism. In this network, attention mechanisms generate multiple representations of tokens, where each representation can refer to a different contextual relationship.

**Figure 1:**
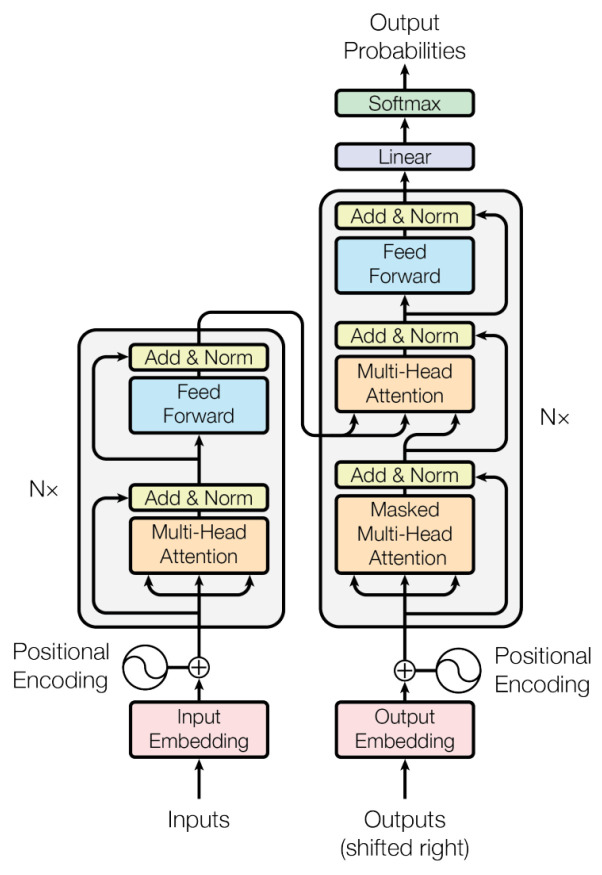
Transformer architecture [19].

Transformers are based on the traditional architecture of Multilayer Perceptron, making massive use of attention mechanisms trained under the encoder–decoder configuration. Figure 1 illustrates the Transformer architecture. The Transformer receives the source and target sequences, concatenated with positional encodings that help the network understand the order between the tokens. The boxes with a light gray background on the left and right represent the encoder and decoder, respectively. Note that the encoder and decoder differ only in the presence of an additional layer of attention in the decoder. The Transformer network considers N stacked encoders–decoders, summarized in Figure 1 by the Nx notation. The embedding produced by the network is taken from its top.

### 2.2. BERT

Bidirectional Encoder Representations from Transformers is a language model based on the transformer architecture [20]. BERT is a language model designed to pre-train bidirectional deep representations from unlabeled text. Its two-way approach aims to model the context to both the right and the left of a given token. Two essential aspects of this approach are that, without substantial changes to its architecture, it can be used (i) pre-trained with or without fine-tuning; and (ii) for tasks that consider individual sentences or sentence pairs (e.g., natural language inference and semantic textual similarity [24]).

In the BERT architecture, there are two essential stages [20]: (i) pre-training; and (ii) fine-tuning. In the first stage, the model is trained on a large unlabeled corpus. While the second one, the model is initialized with the pre-trained data, and all the parameters are fine-tuned using labeled data for specific tasks.

**Figure 2:**
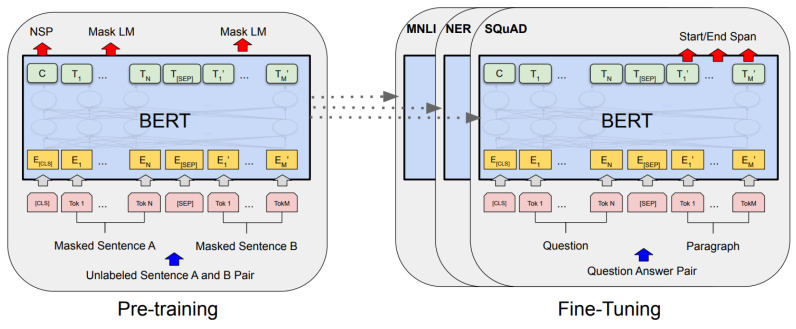
The BERT architecture [20] in a pre-training context (**left**) or fine-tuning for different tasks (**right**).

The architecture of a BERT network can be seen in Figure 2, where the pre-trained version is shown on the left side, and fine-tuned versions adjusted for different tasks are shown on the right. The model is trained using unlabeled data from different tasks in the pre-training stage. In principle, it is possible to use pre-trained BERT models to produce contextual embeddings that can be used for (un)supervised learning tasks. The model is initialized with the pre-trained parameters in the second stage, from fine-tuning to given supervised learning tasks. Then, these parameters are readjusted using data labeled for the task to be solved. Since fine-tuning is performed by task, each task has an individual adjusted model, even if they were initialized with the same pre-trained parameters [20].

To handle various tasks, the representation of BERT input can consist of a single sentence or a pair of sentences. Both possibilities are illustrated at the bottom of the models shown in Figure 2.

### 2.3. Compression of Deep Learning Models

Pre-trained language models (e.g., BERT) have significantly succeeded in various NLP tasks. However, high storage and computational costs prevent pre-trained language models from effectively deploying on resource-constrained devices. To overcome this issue, the compression of deep neural network techniques has been adopted to produce a model with the same robustness as the pre-training models but requires fewer computational resources. Through such a technique, it was possible to design distilled (lightweight) models known as DistilBERT [25].

The compression of the deep neural network is made using knowledge distilling. This compression technique allows a compact model to be trained to reproduce the behavior of a larger model. Dilbert (distilled BERT) is a smaller, faster, general-purpose pre-trained version of BERT that retains nearly the same language comprehension capabilities. The distillation technique [26] consists of training a model based on a larger model, called the teacher, which is used to teach the distilled model, called the student, to reproduce the behavior of the larger model. Thus, DistilBERT is a lightweight model based on the behavior of the original BERT model [25].

The main goal is to produce a smaller model able to reproduce the decisions of the robust bigger model. To do that, it is necessary to approximate the distilled model to the generated function of the bigger model. This function is used to classify a high quantity of pseudo data that show the value of each attribute on the distribution independently [27]. A faster and more compact model trained with pseudo data does not risk present overfitting and will also approximate the learned function from the bigger model [27].

The neural network produces the probability of the classes using a softmax on the output that converts the logit, $z_{i}$, calculated for each class into a probability, $q_{i}$, comparing it with the other logits.

Neural networks typically produce class probability using a softmax output layer that converts the logit, $z_{i}$, calculated for each class into a probability, $q_{i}$, comparing it with the other logits, see Equation (1).

(1) $$q_{i}=\frac{exp(\frac{z_{i}}{T})}{\sum_{j}exp\left(\frac{z_{j}}{T}\right)}$$

where the *T* symbol presented refers to the temperature, typically set to 1; using a more significant value for *T*, a more soft distributed (soft-target) over the classes is obtained.

In the simplest form of distillation, knowledge is transferred to the distilled model by training it with a transfer set. Furthermore, a soft-target distribution is used for each case of the transfer set produced by the larger model with a high value of *T* in its *softmax* [26]. The same *T* with a high value is used to train the distilled model, but temperature 1 is used after training. At low temperatures, distillation pays much less attention to matching the results of the logit function, which are much more negative than the average. Thus, using temperatures more significant than 1, the distilled model extracts more relevant information from the training dataset [26].

### 2.4. BERTimbau: BERT Model for Brazilian Portuguese

It is known that pre-trained models such as BERT have high robustness, but this model is pre-trained with a large amount of English data. To develop a good model for another language such as Brazilian Portuguese, researchers from NeuralMind (https://neuralmind.ai/en/home-en/, accessed on 25 July 2022) developed a BERT model called BERTimbau [28].

To train the model in the Brazilian Portuguese language, the developers used an enormous Portuguese corpus called brWaC, which contains 2.68 billion tokens from 3.53 million documents on the Brazilian webpages [28,29].

Two BERTimbau versions were created: in the first one, BERTimbau Base, the weights were initialized with the checkpoint of Multilingual BERT base, a BERT version trained to 107 languages [30], and trained the model for four days on a TPU (tensor processing unit) v3-8 instance [28]. The second version is called BERTimbau Large; the weights were initialized with the checkpoint of English BERT Large. This version is more significant than the base version and took seven days to train on the same TPU [28]. The version used for evaluation in this article was BERTimbau Base. Additionally, a distilled model from BERTimbau was used and obtained on the HuggingFace Platform (https://huggingface.co/adalbertojunior/distilbert-portuguese-cased, accessed on 25 July 2022).

## 3. Method and Hyperparameter Configuration

This section presents the details of our proposed method for automatic text classification from different languages. The approaches we designed were mainly inspired by the works of Vaswani et al. [19] and Devlin et al. [20], in which attention mechanisms made it possible to track the relations between words across very long text sequences in both forward and reverse directions. Notwithstanding, we explore an extensive dataset from different contexts, including datasets from different languages, specifically English and Brazilian Portuguese, to analyze the performance of the two state-of-the-art models (BERT and DistilBERT).

Our implementation follows the fine-tuning model released in the BERT project [20]. For the multi-class purpose, we use sigmoid cross entropy with logits function to replace the original softmax function, which is appropriate for one-hot classification only. To do this, we first fine-tuned the BERT and DistilBERT, used the aggregating layer as the text embedding, and compared the two models with several selected datasets.

The methodological details are organized into two subsections. The structural steps are the following: Section 3.1 presents the details of the hyperparameter configuration for fine-tuning process, while Section 3.2 presents the environment where the experiments were performed.

### 3.1. Hyperparameter Optimization for Fine-Tuning

In this section, we present the hyperparameter optimization for fine-tuning of our work. All the fine-tuning and evaluation steps performed on each model in this article used the Simple Transformers Library (https://simpletransformers.ai/docs/usage/ accessed on 1 August 2022). Table 1 reports the details of each hyperparameter configuration for fine-tuning process.

The BatchSize is a hyperparameter that controls the number of samples from the training dataset used on each training step. On each step, the predictions are compared with the expected results, an error is calculated, and the internal parameters of the model are improved [31].

The second parameter of Table 1, Epochs, controls the number of times the training dataset will pass through the model during the training process. An epoch has one or more batches [31]. A high number of epochs can make the model overfit, causing it not to generalize, so when the model receives unseen data, it will not make a trustful prevision [32].

**Figure 3:**
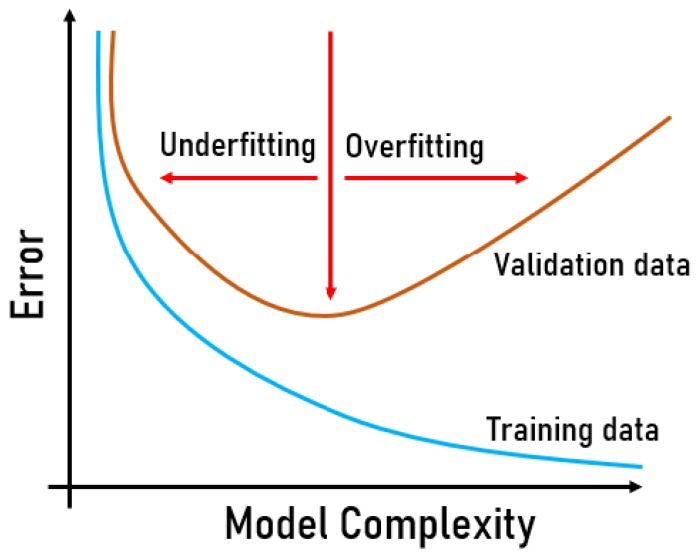
Overfitting example.

Overfitting can be detected in the evaluation step by analyzing the error of the predictions, as in Figure 3. A low number of epochs can also cause underfitting, which means that the models still need more training to learn from the training dataset.

Furthermore, the LearningRate is also related to underfitting or overfitting. This parameter controls how fast the model learns according to the errors obtained. Increasing the learning rate can bring the model from underfitting to overfitting [33].

The Optimizer determines in what measure the weight and the learning rate should be changed in order to reduce the losses of the models. The AdamW is a variant of Adam Optimizer [34]. Adam_epsilon is a parameter used on Adam Optimizer.

The ModelClass refers to the class from the Simple Transformers Library that was used to fine-tune the models. The maximum sequence length parameter refers to the maximum size of the sequence of tokens that can be inputted into the model.

Table 2 presents the hyperparameters of the pre-trained models used in this article for the performance evaluation. The distilled version of the models has six hidden layers, less than the original BERT and BERTimbau models, demonstrating how much smaller the distilled models are. Additionally, the DistilBERT model has 50 million fewer parameters than BERT. The author does not provide the number of parameters of the DistilBERTimbau model.

### 3.2. Implementation

A cloud GPU environment (Google Colab Pro https://colab.research.google.com, accessed on 8 April 2022) was chosen to conduct the fine-tuning process on the models using the datasets selected; the metrics used to evaluate the models were defined. During the fine-tuning process, the (Weights and Biases https://wandb.ai/site, accessed on 8 April 2022) tool was used to monitor each training step and the models’ learning process to detect some overfitting or anything that would bring about poor learning performance.

We trained our models on Google Colab Pro using the hyperparameters described in Table 1 and Table 2. The results were computed and compared between each model to extract information about their performance, and graphics were built to visualize better and compare the results.

Furthermore, the K-fold cross-validation method was used, which consists of splitting the dataset into *n* folders so that every validation set is different from the others. The *K* refers to the number of approximately equal size disjoint subsets, and the fold refers to the number of subsets created. This splitting step is done by randomly sampling cases from the dataset without replacement [35].

**Figure 4:**
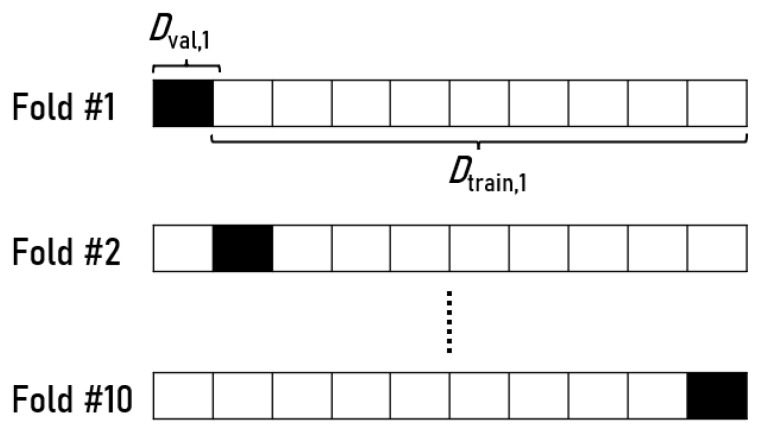
A 10-fold cross-validation example.

Figure 4 represents an example from 10-fold cross-validation. Ten subsets were generated, and each subset is divided into ten parts where nine of them are used to train $D_{train}$ and the other one to evaluate $D_{val}$ the model.

Every evaluation part, $D_{val}$, differs between the subsets. The model is trained, evaluated, and then discarded for each subset or fold, so every part of the dataset will be used for training and evaluation. This allows us to see the potential of the model’s generalization and prevent overfitting [35,36].

To evaluate the models, a 5-fold cross-validation was used. So five subsets were created, and each one was divided into five parts where a fourth of them (80%) are used for the fine-tuning process $D_{train}$, and rest (20%) to evaluate $D_{val}$.

## 4. Case Study

This section has been divided into two parts for a better presentation. The first part, Section 4.1 shows the datasets used in the experiments, while the evaluation metrics are shown in Section 4.2.

### 4.1. Datasets

For this case study, different datasets from the English and Portuguese languages were used. Section 4.1.1 presents the datasets used in the English language, while Section 4.1.2 presents the Brazilian Portuguese ones.

#### 4.1.1. English Language

Three datasets were selected to evaluate the English models. The first one, called the Brexit Blog Corpus [37], contains 1682 phrases provided by a blog associated with Brexit. Those phrases are divided into nine classes, as shown in Table 3. This dataset contains a considerable number of classes and a few examples for each class. It can be seen that this dataset is unbalanced since some classes have less than 50 samples and others more than 200. The choice of an unbalanced dataset was purposeful to evaluate the performance of the chosen models.

The second dataset, called BBC Text, was obtained on Kaggle Platform (https://www.kaggle.com/) and built from BBC News [38], is made up of 2225 comments divided by five classes, as presented in the Table 4. Observing the number of classes and the sample numbers of each class, such a dataset is much more balanced compared to the Brexit Blog Corpus dataset.

The last English dataset selected was the Amazon Alexa Reviews Dataset, also obtained on Kaggle. This dataset contains 3150 feed-backs comments about the Amazon Virtual Assistant Alexa, containing only two classes, positive and negative, presented in Table 5.

This dataset contains much fewer negative samples, but contains only two classes.

#### 4.1.2. Brazilian Portuguese Language

To evaluate the Portuguese models, two datasets were selected. The first, called PorSimples Corpus [39], is a dataset with sentences that passed through different stages of simplifications task. Table 6 contains the stages and the number of sentences produced for each stage of simplification. The Original class contains the original sentences, Natural contains the sentences produced from a Natural stage of simplification of the original sentences, and Strong has the sentences produced on a strong stage of simplification. On each stage of simplification the sentence becomes less complex. In the fine-tuning process, the model will learn the complexity of the sentences and will classify those sentences on three levels, so sentences more complex will be classified as Original, less complex sentences as Natural, and simple sentences as Strong.

The second dataset selected, called Textual Complexity Corpus for School Internships in the Brazilian Educational System Dataset [40], is a dataset that contains texts divided by the stages of the Brazilian educational system. The stages of education are divided into four stages, representing the four classes presented in the Table 7.

### 4.2. Metrics

Four metrics of the evaluation were used to measure the performance of the models. The first one is *accuracy* (Equation (2)), which consists of the number of correct and overall predictions. This metric is the probability that the model predicted the suitable class [41].

(2) $$Accuracy=\frac{\sum CorrectedPredictions}{\sum AllPredictions}$$

The *precision* score (Equation (3)) is used to analyze the proportion of true positives that the model predicted. Precision tells how trustful the model is when predicting a particular class. The calculation is done by dividing the true positives (*TP*) by the sum of true positives (*TP*) and the false positives (*FP*) [41].

(3) $$Precision=\frac{TP}{TP+FP}$$

Additionally, to measure the capability of the model to predict all the positive classes, the *recall* score (Equation (4)) is used. The recall score can be provided by dividing the true positives (*TP*) by the sum of true positives (*TP*) and the false negative (*FN*) [41].

(4) $$Recall=\frac{TP}{TP+FN}$$

The last metric applied in the experiments is the *F*1* Score* (Equation (5)) to measure the performance of the model. This metric uses the precision score (*PS*) and recall score (*RC*) as a weighted average under the concept of harmonic mean [41].

(5) $$F1\;Score=2\times (\frac{PS\times RS}{PS+RS})$$

It is worth mentioning that all metrics presented have their best score as 1 and their worst score as 0.

## 5. Results

This section shows the performance assessment of the BERT, DistilBERT, BERTimbau, and DistilBERTimbau models. For a better presentation, this section was divided into two subsections. The first presented the results of the English language (Section 5.1), and the second presented the results of the Brazilian Portuguese language (Section 5.2).

It is worth mentioning that, after each K-fold iteration, an evaluation is made using the evaluation part of the dataset to measure the score of the fine-tuned model.

### 5.1. English Language

Brexit Blog Corpus was the first dataset evaluated. The BERT model’s results are presented in Table 8 and the DistilBERT model’s results are in Table 9.

The Brexit Blog Corpus dataset obtained relatively low score results for all metrics evaluated, see Table 9. This behavior is expected since the dataset used is unbalanced. That is, many classes and few samples for each class; furthermore, some class has significantly more or fewer samples than others.

Additionally, the score results obtained by the distilled model of BERT are similar to those of its original model BERT. Still, the distilled model took around 47.7% less time on the fine-tuning process than BERT since DistilBERT is a more lightweight model than BERT.

The second English dataset evaluated was the BBC Text. The evaluation score results are presented in Table 10 for the BERT model and in Table 11 for DistilBERT.

Unlike the Brexit Blog Corpus dataset, the BBC Text achieved outstanding score results. It is known that this dataset is balanced, having a good and uniform number of samples for each class. Comparing the two models, the evaluation results are very similar, but the fine-tuning time is around 37.3% lower for DistilBERT compared to BERT.

The last English dataset evaluated was Amazon Alexa Review Dataset. The BERT model’s score result are presented on Table 12 and Table 13 for DistilBERT model.

The Amazon Alexa Reviews dataset reached good results. Analyzing Table 12 and Table 13, it is possible to note that the precision, recall, and F1-score are a little lower than the accuracy score. Those results may occur because the dataset has fewer examples for the negative class and a very high number of samples for the positive class.

The BERT and DistilBERT score results were also very similar when compared. The DistilBERT model took around 52.1% less time to fine-tune when compared to its larger counterpart.

### 5.2. Brazilian Portuguese Language

In order to evaluate the Portuguese model BERTimbau and the distilled version DistilBERTimbau, the first Portuguese dataset selected was the Textual Complexity Corpus for School Internships in the Brazilian Educational System Dataset (TCIE). The BERTimbau score results are presented in Table 14 and the DistilBERTimbau results in Table 15.

The TCIE dataset accomplished good results. Looking over Table 14 and Table 15, it is possible to note that the distilled model had an evaluation score slightly lower than the BERTimbau model on every metric, but the fine-tuning process took around 21.5% longer on BERTimbau than the distilled version.

The second Portuguese dataset used was the PorSimples Corpus. For this dataset, the parameters used on the other datasets presented in Table 1 caused overfitting. A lower number of the learning rate hyperparameter was used to correct this issue, 0.000001 instead of 0.00004. This reduces the model’s learning speed, solving the overfitting issue. The BERTimbau results are presented in Table 16 and the DistilBERTimbau evaluation score results are presented in Table 17.

The evaluation result with this dataset did not achieve very high scores in both the BERTimbau and DistilBERTimbau models. These low results may be explained because, on the PorSimples Corpus dataset, some sentences are similar to the others when passing through the simplifications process, so similar sentences are presented in each dataset class. Hence, the model has more challenges when learning the class differences. Additionally, the BERTimbau model took around 49.2% more time than the distilled model to the fine-tuning process. Furthermore, the high time results presented in Table 16 and Table 17 were expected since this dataset has 11,944 samples, many more when compared to the other datasets.

Table 18 contains the size of the models generated after the fine-tuning process for each dataset. Analyzing the results, it is possible to identify that the distilled models produced models around 40% smaller than their larger counterparts.

An important observation is that on every evaluation, the scores reached on every k-fold iteration had very similar results, which show the model’s generalization capability.

**Figure 5:**
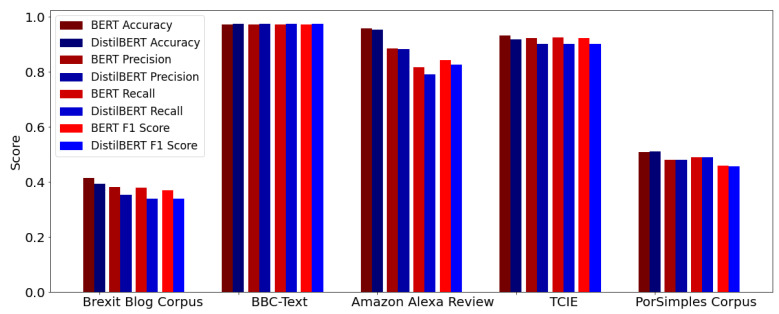
Bar plot comparing BERT and DistilBERT’s model scores.

The barplot presented in Figure 5 contains the arithmetic mean of each scoring metric on each k-fold iteration. In this figure, the red bars refer to BERT/BERTimbau models, and the blue ones to DistilBERT/DistilBERTimbau models.

As we can see, the score recorded by the distilled models is very similar to the ones scored by the original models. This shows the power of the compression of deep learning models technique, which produces smaller models, requires fewer computation resources, and has almost the same power as the original models.

## 6. Discussion

Analyzing the results presented in Section 5 and Figure 5, the scores recorded by the distilled models are very similar to the ones scored by the original models. In our experiments, they were around 45% faster in the fine-tuning process, about 40% smaller, and also preserving about 96% of the language comprehension skills performed by BERT and BERTimbau. It is worth noting that these results are similar to the results presented on [25], where the DistilBERT models were 40% smaller, 60% faster, and retained 97% of BERT’s comprehension capability.

The work presented in [42] compared BERT, DistilBERT, and other pre-trained models for emotion recognition and also achieved similar score results on BERT and DistilBERT. Furthermore, the DistilBERT model was the fastest one. These results presented in that work and also in the literature show the power of the compression of deep learning models technique, which produces smaller models, requires fewer computation resources, and has almost the same power as the original models.

Another critical point we can highlight in Figure 5 is the importance of the quality of the datasets to produce a good predicted model. In two unbalanced datasets, such as Brexit Blog Corpus and PorSimples Corpus, the accuracy was low against the other balanced datasets. The Amazon Alexa Reviews achieve good accuracy, but lower precision, recall, and F1 score since this dataset has a low number of negative samples.

Other pre-trained models have been widely developed for other languages such as BERTino [43], an Italian DistilBERT, and CamemBERT [44] for the French language based on the RoBERTa [45] model, a variation of the BERT model. The main goal of pre-trained models is to remove the necessity of building a specific model for each task and to improve the necessity of developing a pre-trained model for each language, bigger models that understand multiple languages have been developed such as BERT Multilingual [30] and also GPT-3 [46]. Still, those models are trained with more data than BERT for specific languages, especially GPT-3, and should require more computational resources.

## 7. Conclusions

Inspired by a state-of-the-art language representation model, this paper analyzed two state-of-the-art models, BERT and DistilBERT, for text classification tasks for both English and Brazilian Portuguese. These models have been compared with several selected datasets. The experiment results showed that the compression of neural networks responsible for the generation of the DistilBERT and DistilBERTimbau produce models around 40% smaller and take around 45% (our experiments ranged from 21.5% to 66.9%) less time for the fine-tuning process. In other words, compression models require fewer computational resources, which did not significantly impact the model’s performance. Thus, the lightweight models allow being executed with low computational resources and with the performance of their larger counterparts. In addition, the distilled models preserve about 96% of language comprehension skills for balanced datasets.

Some extensions of our future work can be highlighted: *(i)* other robust models are being widely studied and developed, such as in [47] and GPT-3 [46], which can be evaluated and compared with the models mentioned in this work; and *(ii)* perform task classification for non-Western languages (e.g., Japanese, Chinese, and Korean).

In closing, the experiment results show how robust the Transformer architecture is and the possibility of using it for more languages than English, such as the Brazilian Portuguese models studied in this work.

## Figures and Tables

**Table 1:** Hyperparameters of the fine-tuned model.

Hyperparameter	Value
Batch size	8
Epochs	10
Learning rate	0.00004
Optimizer	AdamW
Adam_epsilon	0.00000001
Model class	Classification model
Maximum sequence length	128

**Table 2:** Model hyperparameters.

Hyperparameter	BERT Base [20]	DistilBERT Base [25]	BERTimbau Base ${}^{1}$	DistilBERTimbau Base ${}^{2}$
Hidden layers	12	6	12	6
Total parameters	110 M	66 M	110 M	-

^1^https://huggingface.co/neuralmind/bert-base-portuguese-cased accessed on 1 August 2022. ^2^ https://huggingface.co/adalbertojunior/distilbert-portuguese-cased accessed on 1 August 2022.

**Table 3:** Brexit Blog Corpus dataset.

ID	Classes	Number of Examples
0	agreement/disagreement	50
1	certainly	84
2	contrariety	352
3	hypothetically	171
4	necessity	204
5	prediction	252
6	source of knowledge	287
7	tact/rudeness	44
8	uncertainty	196
9	volition	42
	Total	1682

**Table 4:** BBC Text dataset.

ID	Classes	Number of Examples
0	business	510
1	entertainment	386
2	politics	417
3	sport	511
4	tech	401
	Total	2225

**Table 5:** Amazon Alexa Reviews dataset.

ID	Classes	Number of Examples
0	positive	2893
1	negative	257
	Total	3150

**Table 6:** PorSimples Corpus dataset.

ID	Classes	Number of Examples
0	Original	2907
1	Natural	4066
2	Strong	4971
	Total	11,944

**Table 7:** Textual Complexity Corpus for School Internships in the Brazilian Educational System dataset.

ID	Classes	Number of Examples
0	Elementary School—I	297
1	Elementary School—II	325
2	High School	628
3	University Education	826
	Total	2076

**Table 8:** BERT—Brexit Blog Corpus dataset.

K-Fold	Accuracy	Precision	Recall	F1 Score	Evaluation Loss	Training Time	Evaluation Time
1	0.3650	0.3345	0.3290	0.3279	2.558	395	12
2	0.4214	0.4270	0.4017	0.4068	2.497	384	12
3	0.4107	0.3421	0.3495	0.3433	2.516	385	13
4	0.4286	0.3754	0.4223	0.3761	2.320	391	11
5	0.4405	0.4217	0.3909	0.3991	2.256	390	10

**Table 9:** DistilBERT—Brexit Blog Corpus dataset.

K-Fold	Accuracy	Precision	Recall	F1 Score	Evaluation Loss	Training Time	Evaluation Time
1	0.3561	0.3078	0.2971	0.2981	2.546	205	12
2	0.4154	0.3650	0.3557	0.3494	2.298	208	10
3	0.4137	0.3687	0.3606	0.3537	2.373	205	11
4	0.3750	0.3807	0.3442	0.3506	2.421	199	11
5	0.4048	0.3480	0.3352	0.3378	2.357	201	11

**Table 10:** BERT—BBC Text dataset.

K-Fold	Accuracy	Precision	Recall	F1 Score	Evaluation Loss	Training Time	Evaluation Time
1	0.9753	0.9742	0.9747	0.9743	0.2062	435	10
2	0.9820	0.9826	0.9810	0.9816	0.1470	438	12
3	0.9820	0.9808	0.9821	0.9812	0.1116	450	13
4	0.9685	0.9685	0.9694	0.9689	0.2923	432	13
5	0.9573	0.9608	0.9540	0.9565	0.3191	438	13

**Table 11:** DistilBERT—BBC Text dataset.

K-Fold	Accuracy	Precision	Recall	F1 Score	Evaluation Loss	Training Time	Evaluation Time
1	0.9685	0.9680	0.9685	0.9681	0.2478	278	11
2	0.9685	0.9690	0.9705	0.9694	0.2098	269	12
3	0.9775	0.9747	0.9763	0.9754	0.2041	266	13
4	0.9753	0.9758	0.9763	0.9760	0.2039	266	13
5	0.9888	0.9877	0.9879	0.9876	0.0955	277	13

**Table 12:** BERT—Amazon Alexa Review dataset.

K-Fold	Accuracy	Precision	Recall	F1 Score	Evaluation Loss	Training Time	Evaluation Time
1	0.9651	0.8998	0.8377	0.8656	0.2050	665	12
2	0.9587	0.9191	0.7766	0.8304	0.2890	663	13
3	0.9508	0.8299	0.8109	0.8201	0.3314	663	14
4	0.9619	0.8523	0.8667	0.8593	0.2755	666	14
5	0.9508	0.9305	0.7919	0.8443	0.3640	666	14

**Table 13:** DistilBERT—Amazon Alexa Review dataset.

K-Fold	Accuracy	Precision	Recall	F1 Score	Evaluation Loss	Training Time	Evaluation Time
1	0.9603	0.8902	0.8065	0.8423	0.2431	324	09
2	0.9492	0.8725	0.8016	0.8323	0.3847	314	10
3	0.9413	0.8803	0.7228	0.7763	0.4456	319	11
4	0.9571	0.8369	0.8298	0.8333	0.3127	317	11
5	0.9619	0.9344	0.7966	0.8469	0.2698	318	11

**Table 14:** BERTimbau—TCIE dataset.

K-Fold	Accuracy	Precision	Recall	F1 Score	Evaluation Loss	Training Time	Evaluation Time
1	0.9351	0.9232	0.9280	0.9233	0.3477	418	12
2	0.9325	0.9227	0.9194	0.9206	0.4158	408	12
3	0.9301	0.9172	0.9193	0.9181	0.3752	421	13
4	0.9422	0.9375	0.9395	0.9384	0.3229	414	14
5	0.9253	0.9211	0.9168	0.9186	0.5360	241	13

**Table 15:** DistilBERTimbau—TCIE dataset.

K-Fold	Accuracy	Precision	Recall	F1 Score	Evaluation Loss	Training Time	Evaluation Time
1	0.9135	0.9060	0.9087	0.9072	0.5106	299	13
2	0.9277	0.9137	0.9180	0.9148	0.4370	289	15
3	0.9253	0.9025	0.9051	0.9024	0.3878	291	14
4	0.9181	0.9026	0.8990	0.9006	0.4631	307	15
5	0.9036	0.8822	0.8783	0.8797	0.6200	308	14

**Table 16:** BERTimbau—PorSimples Corpus dataset.

K-Fold	Accuracy	Precision	Recall	F1 Score	Evaluation Loss	Training Time	Evaluation Time
1	0.5184	0.4948	0.5056	0.4690	0.9385	2162	25
2	0.5126	0.4824	0.4881	0.4679	0.9665	2159	25
3	0.5008	0.4737	0.4912	0.4518	0.9543	2170	26
4	0.5021	0.4813	0.4892	0.4634	0.9647	2173	24
5	0.5050	0.4681	0.4718	0.4483	0.9768	2198	27

**Table 17:** DistilBERTimbau—PorSimples Corpus dataset.

K-Fold	Accuracy	Precision	Recall	F1 Score	Evaluation Loss	Training Time	Evaluation Time
1	0.5038	0.4797	0.4801	0.4520	0.9635	1099	15
2	0.5067	0.4697	0.4837	0.4449	0.9559	1098	17
3	0.5226	0.4889	0.5030	0.4687	0.9311	1095	18
4	0.5251	0.4892	0.4968	0.4721	0.9428	1126	18
5	0.5008	0.4794	0.4853	0.4446	0.9746	1103	17

**Table 18:** Model Size.

Dataset	BERT	DistilBert
Amazon Alexa Review	413.3 MB	251 MB
BBC Text	413.3 MB	251 MB
Brexit Blog Corpus	413.3 MB	251 MB
TCIE	415.6 MB	253.4 MB
PorSimples Corpus	415.6 MB	253.3 MB

## Data Availability

All the datasets used in this work can be found on the links below: Brexit Blog Corpus dataset-https://snd.gu.se/en/catalogue/study/snd1037#dataset; BBC Text dataset-https://www.kaggle.com/code/yufengdev/bbc-text-categorization/data; Amazon Alexa Reviews dataset-https://www.kaggle.com/datasets/sid321axn/amazon-alexa-reviews; PorSimples Corpus dataset-https://github.com/sidleal/porsimplessent; Textual Complexity Corpus for School Internships in the Brazilian Educational System dataset-https://github.com/gazzola/corpus_readability_nlp_portuguese.
